# A modified Edmonton Symptom Assessment Scale for symptom clusters in radiation oncology patients

**DOI:** 10.1002/cam4.1125

**Published:** 2017-08-04

**Authors:** Peter A. S. Johnstone, Jae Lee, Jun‐Min Zhou, Zhenjun Ma, Diane Portman, Heather Jim, Hsiang‐Hsuan Michael Yu

**Affiliations:** ^1^ Radiation Oncology H. Lee Moffitt Cancer Center & Research Institute Tampa Florida; ^2^ Health Outcomes & Behavior H. Lee Moffitt Cancer Center & Research Institute Tampa Florida; ^3^ Biostatistics and Bioinformatics H. Lee Moffitt Cancer Center & Research Institute Tampa Florida; ^4^ Supportive Care Medicine Departments H. Lee Moffitt Cancer Center & Research Institute Tampa Florida

**Keywords:** ESAS, patient‐reported outcomes, radiation oncology, symptom clusters, symptom management

## Abstract

Patient‐reported outcomes regarding symptom burden may provide valuable information in addition to physician assessment. Systematic collection of patient‐reported outcomes may be an important metric to identify unmet needs and improve quality of patient care. To understand common symptoms of patients seen in radiation oncology clinic, we examined the prospectively collected modified Edmonton Symptom Assessment Scale (ESAS‐r) data to explore symptom clusters. Our clinic established use of a modified Edmonton Symptom Assessment Scale in August 2015. All outpatients presenting for radiation oncology services completed the form at each clinic visit. Symptom clusters are defined by two or more symptoms that are interrelated and occur simultaneously with a high degree of predictability. A sample of 916 de‐identified surveys was assessed statistically using principal component analysis (PCA) with varimax rotation to determine independent clustering between the symptoms queried. We found four major clusters of symptoms: Tiredness (tired, drowsiness; PC1), Loss of Appetite (nausea, lack of appetite; PC2), Low Well‐Being (overall & spiritual well‐being; PC3), and Depression (depression, anxiety; PC4). These accounted for 46%, 9.2%, 7.6%, and 7% of total variance, respectively. Internal consistency using Cronbach's alpha was 0.87, 0.7, 0.82, and 0.87, respectively. The most frequent write‐in item was itchiness, present in 24% of the 148 patients responding. Assessment of patients seen in a large radiation oncology clinic revealed several symptom clusters. {Tiredness and drowsiness} represents a major symptom cluster. Itchiness may be underrecognized.

## Introduction

There is increasing recognition of the importance of assessing patient‐reported symptoms as part of clinical care. Recent randomized trials of clinic‐based symptom monitoring have found that it is associated with reduced symptom severity, better quality of life, and reduced hospitalization and emergency room visits relative to usual care 1, 2. Moreover, patients and providers report high satisfaction with symptom monitoring 1, 2, indicating that it results in improved communication about symptoms with little to no additional burden. Improved care satisfaction and quality of life are important metrics for delivery of optimal care quality.

The Edmonton Symptom Assessment Scale (ESAS) and other patient self‐report tools have facilitated a growing body of literature regarding symptomatology in palliative care patients, including patients with advanced cancer 3, 4. As these patients often experience multiple symptoms that affect quality of life and outcomes, recent symptom management literature has focused on identifying and managing symptom clusters 5, 6. A commonly applied definition of symptom cluster is two or more symptoms that are interrelated and occur simultaneously with a high degree of predictability 7, 8.

Most data regarding patient‐reported symptoms come from research studies rather than standard clinical practice. Research participants tend to be younger and healthier than most cancer patients 9, 10, 11. For this reason symptom data collected as part of a research study may not accurately reflect the experience of the larger patient population, particularly patients with advanced cancer. Uniquely in this report, we report the feasibility and results of a broad approach to using the ESAS tool in a busy clinical environment: the general population of radiation oncology patients. These data are important because they may be used to adequately resource clinics. For instance, prior studies have been limited to patients receiving radiation in a palliative care *milieu* (3), or in the setting of advanced cancer (6). Such results are generalizable to the population involved, but are less useful when supporting personnel funding decisions for a busy clinic. For instance, social work or mental health support is budgeted on the entire clinic population and includes consults, follow‐ups, and patients on treatment. There are no other studies in the literature to our knowledge describing patient‐reported outcomes broadly in such a population.

## Materials and Methods

In August 2015, Moffitt Cancer Center introduced a clinical initiative within the Radiation Oncology Department. This project involved distributing patient report forms to each patient being seen in the radiation oncology clinic. This included new and follow‐up patients as well as those being seen weekly while on treatment. Each patient was provided a blank carbon form with the ESAS questionnaire. Medical assistants provided guidance as needed to facilitate completion of the forms. The clinical practitioners reviewed and verified completed forms with the patients. ESAS assesses nine common symptoms with option of adding a tenth symptom; each symptom is rated on an 11‐point (0–10) numerical rating scale. ESAS‐r retains the core elements of the ESAS, with revisions focusing on symptom assessment time frame, terminology, item order, and format 12, 13. ESAS‐r‐CS contains further modification to include constipation and sleep disturbance, and addition of a spiritual well‐being domain to the ESAS‐r‐CS elements is termed ESAS‐r‐CSS which is the form used for this study 14. The ESAS‐r‐CSS retains a blank scale to fill in issues not otherwise covered. These forms were then collected and scanned into the electronic medical record. Although NCCN guidelines currently recommend intervention for symptoms rated as 4 or greater on a 0–10 scale 15, a value of 7 or above was decided as the cutoff for intervention to ensure that resources were allocated to the patients with the greatest need 16.

Approval for this retrospective review was obtained from the Moffitt Cancer Center Institutional Review Board. A sample of 916 forms was retrieved and information collated. These data with the self‐reported scores for each domain were transferred to an Excel spreadsheet.

We consider this sample to represent the entire constellation of patients for whom the radiation oncology program provides services. No stratification was made for type of cancer, degree of metastatic disease (if present), or performance status. Furthermore, this large sample size allowed us to carefully examine relationships among symptoms.

Results were expressed as mean, median, and standard deviation for quantitative variables and as proportions for categorical variables. If more than one number was marked for any symptom, the mean of those numbers was used. Blank fields were noted as such. Correlative statistics among symptoms were performed using Pearson's correlation analysis by SAS 9.4.

To evaluate the presence of symptom clusters, a hierarchical clustering analysis was first performed. Next, a principal component analysis with varimax rotation was performed on the ESAS symptom data. The internal consistency of the derived clusters was assessed with the overall standardized Cronbach's coefficient alpha. Biplots were generated using S‐PLUS software for visualization of magnitude and direction of each variable's contribution to the components, as well as how each observation was represented in terms of those components.

## Results

Demographic information on this patient cohort is shown in Table 1. Data from 916 unique encounters were accessed and tabulated. Of these, only 62 encounters (6.77%) had no symptoms reported on the ESAS‐r‐CSS. Frequency of the symptoms is listed in Table 2. Fatigue was the most common symptom (76%), and had the highest mean value in symptomatic patients. Of the forms reporting a single symptom, it was most commonly “tired” or “difficulty sleeping” (each 19% of 21 single response surveys). In Table 2, symptom severity is categorized as mild for scores <4, moderate for scores from 4 to <7, and severe for scores >7. On 148 forms (16.16%) the patients wrote symptoms into the blank space in block 13 of the ESAS‐r‐CSS. The most frequently reported in this item was itchiness (23%). Frequency of other responses is at Table 3.

**Table 1:** Characteristics of patients completing 916 questionnaires

Characteristics		Data (%)
Age	Median	64 years
	Range	21–93 years
Gender	Female	373 (41.9)
	Male	517 (58.1)
Primary disease site	Breast	124 (13.9)
	Cutaneous	77 (8.7)
	Endocrine	1 (0.1)
	GI	68 (7.6)
	GU	154 (17.3)
	Gyn	34 (3.8)
	H&N	161 (18.1)
	Heme	25 (2.8)
	Neuro	54 (6.1)
	Sarcoma	50 (5.6)
	Thoracic	142(6)

**Table 2:** Prevalence, mean, and median ESAS+ scores

Symptom	Total (n)	Prevalence of score n (%)			Mean (SD)	Median (range)
		0–2.9	3–6.9	7–10		
Pain	903	563 (62.3)	251 (27.8)	89 (9.9)	3.87 (2.39)	3 (1–10)
Tired	911	401 (44)	355 (39)	155 (17)	4.39 (2.53)	4 (1–10)
Drowsiness	910	556 (61.1)	257 (28.2)	97 (10.7)	3.91 (2.48)	3 (1–10)
Nausea	913	780 (85.4)	96 (10.5)	37 (4.1)	3.44 (2.48)	3 (1–10)
Lack of appetite	908	669 (73.7)	165 (18.2)	74 (8.1)	4.22 (2.57)	4 (1–10)
Shortness of breath	910	713 (78.4)	143 (15.7)	54 (5.9)	3.6 (2.45)	3 (1–10)
Depression	913	700 (76.7)	152 (16.6)	61 (6.7)	3.7 (2.49)	3 (1–10)
Anxiety	911	626 (68.7)	203 (22.3)	82 (9)	3.81 (2.46)	3 (1–10)
Overall well‐being	910	492 (54.1)	319 (35.1)	99 (10.9)	3.86 (2.38)	3 (1–10)
Spiritual well‐being	894	652 (72.9)	157 (17.6)	85 (9.5)	3.81 (2.78)	3 (1–10)
Constipation	913	705 (77.2)	161 (17.6)	47 (5.1)	3.66 (2.34)	3 (1–10)
Difficult sleeping	915	523 (57.2)	254 (27.8)	138 (15.1)	4.28 (2.66)	4 (1–10)

**Table 3:** Symptoms written in to the form's blank space (*n* = 148)

Symptom	Total (n)	Prevalence (%)	Mean (SD)	Median (range)
Itchiness	34	23	3.97 (2.26)	3.5 (1–8)
Swallowing issues	9	6.1	7.06 (2.65)	7.5 (3–10)
Urinary issues	6	4.1	4.00 (1.9.0)	4.0 (2–7)
Hiccups	4	2.7	8.75 (1.26)	9 (7–10)
Headache	3	2	6.00 (2.65)	5 (4–9)
Diarrhea	4	2.7	7.25 (2.99)	8 (3–10)
Various	88	59.5	5.99 (2.54)	6 (0–10)

**Figure 1:**
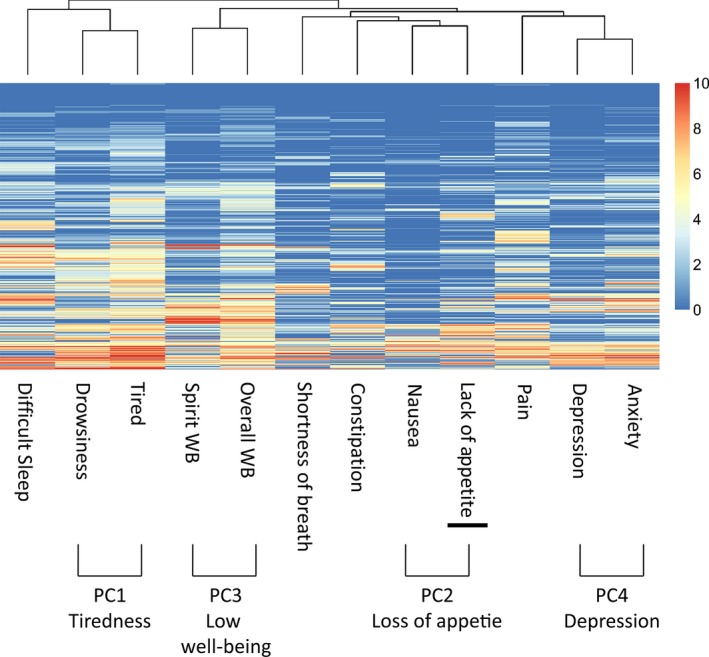
Hierarchical cluster analysis among symptoms.

**Figure 2:**
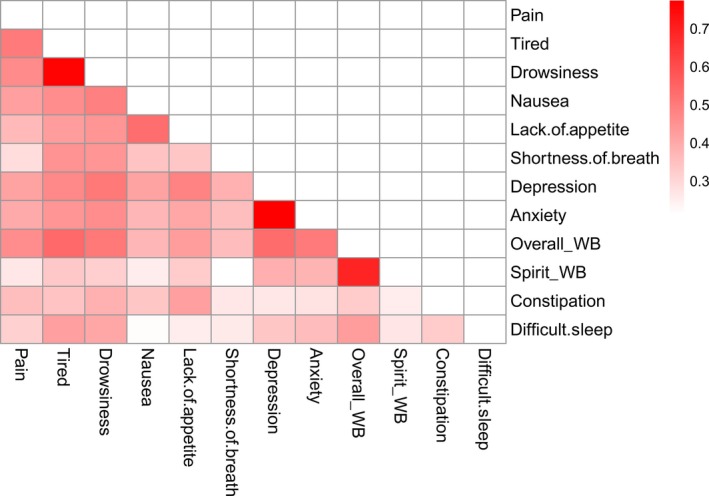
Pearson correlation of symptoms.

The unsupervised hierarchical clustering identified four major clusters of symptoms: Tiredness (tired, drowsiness; PC1), Loss of Appetite (nausea, lack of appetite; PC2), Low Well‐Being (overall & spiritual well‐being; PC3), and Depression (depression, anxiety; PC4) Clusters (Fig. 1). Figure 2 shows the Pearson correlation coefficients among 12 symptoms. Strong correlations were found between tired and drowsiness, depression and anxiety, overall and spiritual well‐being, nausea and lack of appetite, which were main symptom variables for the four clusters above. A principal component analysis further confirmed that these clusters could be described with four main principal components as shown in Table 4. These four principal components accounted for 46%, 9.2%, 7.6%, and 7% of total variance, respectively. In particular, tiredness and drowsiness symptoms were highly associated with each other and clustered together in PC1, which accounted for 46% of total variance of patient symptom scores; the other three symptom clusters accounted for 9.2%, 7.6%, and 7% of total variance, respectively. Therefore, we concluded that this cluster represented a major symptom cluster in our ESAS data.

**Table 4:** Factor loadings and final communality of symptoms

Symptom	Component 1	Component 2	Component 3	Component 4	Final communality
Tired	**0.80**	0.26	0.23	0.19	0.80
Drowsiness	**0.75**	0.32	0.17	0.22	0.74
Pain	0.40	0.35	0.22	0.21	0.37
Shortness of breath	0.38	0.29	0.14	0.23	0.31
Difficult sleeping	0.35	0.17	0.29	0.17	0.27
Overall well‐being	0.31	0.21	**0.86**	0.23	0.93
Nausea	0.30	**0.60**	0.11	0.20	0.51
Constipation	0.28	0.46	0.18	0.08	0.33
Anxiety	0.26	0.21	0.25	**0.73**	0.71
Depression	0.24	0.27	0.25	**0.82**	0.86
Lack of appetite	0.17	**0.71**	0.19	0.23	0.62
Spirit well‐being	0.13	0.17	**0.67**	0.19	0.53
% of Variance	45.99	9.15	7.61	7.04	
Cronbach's alpha	0.87	0.70	0.82	0.87	

Clusters noted in bold.

**Figure 3:**
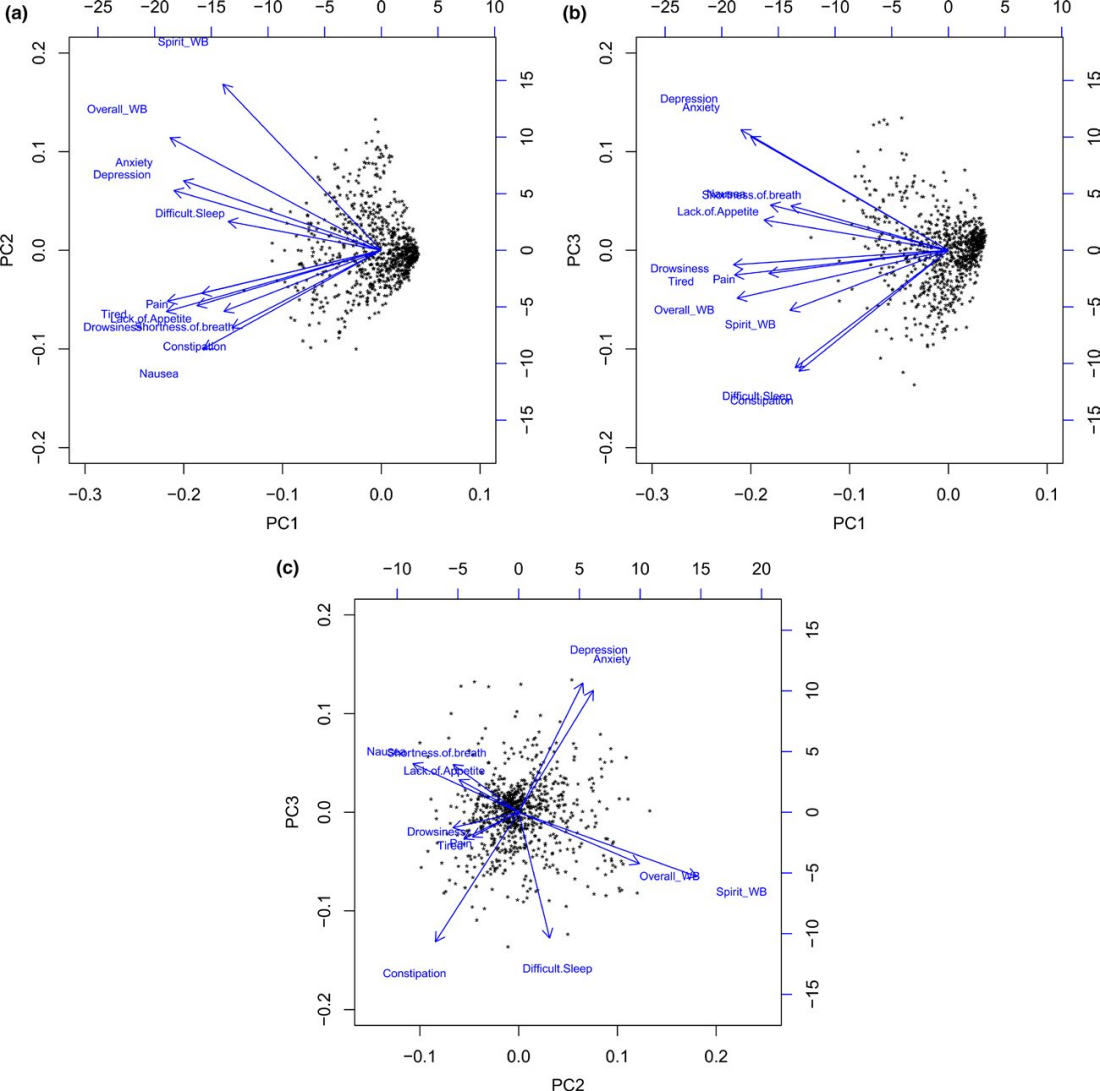
Biplot among three principal components or clusters, depicting the planes of a three‐dimensional model.

Internal consistency using Cronbach's alpha coefficient was 0.87, 0.7, 0.82, and 0.87, respectively. Biplots were depicted to show symptom score distributions based on these principal components in a three‐dimensional space (Fig. 3).

## Discussion

Our study confirms that ESAS‐r‐CSS can be well utilized by the large spectrum of radiation oncology outpatients. Using this validated symptom assessment tool, we were able to collect patient‐reported outcomes for quantitative assessment of symptom burden. Symptom assessment by physicians does not always accurately reflect symptoms experienced by the patients. Symptom screening tools such as ESAS have been shown to improve communication and care quality, and there is increasing effort to integrate them in clinical practice.

No prior study to our knowledge has examined patient‐reported symptoms collected as part of clinical care in the broad radiation oncology population: before, during, and after treatment, and then looked for clustering in symptom self‐reports. Our data yield the following clusters: {tired, drowsiness}, {nausea, lack of appetite}, {overall & spiritual well‐being}, and {depression, anxiety}.

While first developed for a cancer population with advanced disease 3, ESAS is utilized for patients at various stage of illness 17. Studies have shown evidence of validity in cancer patients with both advanced disease 18, 19 and earlier in the course of disease 4, 20. ESAS may also be used for symptom monitoring over time in nonpalliative patients seen by oncology in both inpatient and outpatient settings 21. Studies have employed the ESAS across the trajectory of cancer care from survivorship to patients receiving treatment to patients with advanced disease and at the end of life 22, 23, 24, 25. Studies based on ESAS have revealed discrete symptom clusters in patients with bone and brain metastases 26, 27. In the first case, Chow and colleagues surveyed 518 patients with bone metastases and documented three symptoms accounting for 66% of the total variance of symptoms: {fatigue, pain, drowsiness, poor sense of well‐being}, {anxiety, depression}, and {shortness of breath (SOB), nausea, poor appetite}. Internal reliability for each of these varied between 0.61 and 0.81 27. The second study revealed slightly different clustering of symptoms 28. Comparison of data from both manuscripts shows more variability in the latter report which is not surprising given the markedly smaller number of responses.

Studies of ESAS symptom cluster changes over time have been performed in radiation oncology patients, but only in specific circumstances or populations. For instance, Chow and colleagues prospectively documented a decrement in fatigue, drowsiness, and appetite scores after whole brain radiation therapy 28. Similarly, data in the bone metastasis study noted above reveal that pain became less of an issue after palliative radiotherapy 26. It is also possible that certain clinical or demographic factors may correlate with symptoms or symptom clusters. A limitation of our study is that the data have been de‐identified prior to analysis, and therefore we are not able to explore possible correlation. It is likely a subject of further study whether “depression” and “anxiety”, as recognized by ESAS‐r‐CSS, are sufficiently unique. Cronbach's alpha coefficient for this cluster in the bone metastasis study was 0.81 26, and was 0.74 in the brain metastasis cohort 28. In each case it was the most correlated cluster. In our data, Cronbach's alpha was 0.87. There is an ongoing debate in the literature about this point with proponents 29, 30 and opponents of the tool 31. Nevertheless, the ESAS can be used as a screening tool for a mood disorder; patients endorsing high levels of these symptoms should be referred to psychosocial services for a more thorough evaluation and appropriate intervention.

Limitations of this study include the fact that some patients may have contributed data to the sample on multiple separate dates, as the unit of measure was by encounter rather than by patient. For instance, patients are seen weekly while on treatment and fill out multiple forms over time. This was not specifically addressed statistically given results of a recent systematic review performed to examine the composition, longitudinal stability, and consistency across methodologies of common symptom cluster, as well as their common predictors and outcomes 3. These data showed that symptom cluster found at baseline were unstable or of mixed stability over time, regardless of the type of statistical analysis used 3. We did note on data entry that there were few episodes of such multiple reports from individual patients (patient identifiers were not tracked once the data were collected), and we assumed that these few cases would not be perfectly congruent over time.

There are several analysis issues worth mentioning. First, statistical rules are generally subjective for determining the number of principal components, but it is common to retain top principal components to cumulatively explain 60–80% variation of the whole data. Therefore, we chose four PCs, each of which explained at least >7% of variance, and cumulatively explained ~70% of the total variance.

To evaluate the presence of symptom clusters, a hierarchical clustering analysis was performed first followed by principal component analysis. In our principal component analysis, patients with any missing variable values were excluded from the analysis. However, we found that the proportion of these excluded patients due to missing data was very small with 22 excluded cases of total 916 cases (2.46%). Therefore, we believe that missing data do not likely contribute to significant bias in our analysis. Note that we performed a k‐nearest‐neighbor algorithm as an imputation method for missing data for drawing the Biplot plots. As the ESAS symptom scores were collected with a standardized range between 0 and 10, we did not find influential outliers that might have significantly affected our analysis results.

## Conclusion

In this assessment of patient‐reported symptoms using a standard ESAS survey (ESAS‐r‐CSS) in a large radiation oncology department, several symptom clusters present. {Tiredness and drowsiness} represents a major symptom cluster. Itchiness may be underrecognized in this population. In addition to being able to use these data for observational analyses, appropriate intervention for moderate‐to‐high symptom burden can be triggered in a timely manner. Given this successful implementation, we now are investigating using online solutions to allow the data to be collected in discrete form and instantly uploaded to our institutional electronic medical record. We plan to explore symptom burden in discrete subpopulations of our patients (e.g., those at end of life, young adults) to further clarify their unique needs. Ultimately we plan to generate a dashboard at the provider level so that patients requiring and receiving special symptom management will be better tracked.

## Conflict of Interest

Dr. Johnstone reports personal fees from Novocure and travel support from View Ray. Dr. Lee has nothing to disclose. Dr. Ma has nothing to disclose. Dr. Zhou has nothing to disclose. Dr. Portman has nothing to disclose. Dr. Jim has nothing to disclose. Dr. Yu reports personal fees from UpToDate, Inc.
